# Dual role of the colonization factor CD2831 in *Clostridium difficile* pathogenesis

**DOI:** 10.1038/s41598-019-42000-8

**Published:** 2019-04-03

**Authors:** Vanessa Arato, Gianmarco Gasperini, Fabiola Giusti, Ilaria Ferlenghi, Maria Scarselli, Rosanna Leuzzi

**Affiliations:** 1 Glaxo Smith Kline Vaccines, Via Fiorentina 1, 53100 Siena, Italy; 2 University of Padova, Department of Biomedical Sciences, 35131 Padua, Italy; 3 GSK Vaccines Institute for Global Health (GVGH), Via Fiorentina 1, 53100 Siena, Italy

## Abstract

*Clostridium difficile* is a Gram-positive, anaerobic bacterium and the leading cause of antibiotic-associated diarrhea and pseudomembranous colitis. *C. difficile* modulates its transition from a motile to a sessile lifestyle through a mechanism of riboswitches regulated by cyclic diguanosine monophosphate (c-di-GMP). Previously described as a sortase substrate positively regulated by c-di-GMP, CD2831 was predicted to be a collagen-binding protein and thus potentially involved in sessility. By overexpressing CD2831 in *C. difficile* and heterologously expressing it on the surface of *Lactococcus lactis*, here we further demonstrated that CD2831 is a collagen-binding protein, able to bind to immobilized collagen types I, III and V as well as native collagen produced by human fibroblasts. We also observed that the overexpression of CD2831 raises the ability to form biofilm on abiotic surface in both *C. difficile* and *L. lactis*. Notably, we showed that CD2831 binds to the collagen-like domain of the human complement component C1q, suggesting a role in preventing complement cascade activation via the classical pathway. This functional characterization places CD2831 in the Microbial Surface Components Recognizing Adhesive Matrix Molecule (MSCRAMMs) family, a class of virulence factors with a dual role in adhesion to collagen-rich tissues and in host immune evasion by binding to human complement components.

## Introduction

The sporogenic, anaerobic rod *Clostridium difficile* is a Gram-positive enteropathogen causing *C. difficile* Infection (CDI). CDI ranges from mild and self-limiting diseases to a severe, life-threatening pseudomembranous colitis, toxic megacolon, sepsis and death^1,2^. Transmission of *C. difficile* is mediated by spores, which being resistant to oxygen and to many disinfectants and sterilants, persist in the environment^3^. A dramatic increase in the incidence and mortality of CDI has been observed worldwide^4^. Two large glycosylating exotoxins, TcdA and TcdB, are the determinants of clinical symptoms by causing the loss of the intestinal membrane integrity and cell death^5^. CDI is most frequently associated with prolonged antibiotic treatment which, by depleting the normal microbiota of the gut, allows *C. difficile* to proliferate in the colon and to produce toxins^6^.

Adhesion to the host epithelial cells is the first crucial step to the bacterial settlement in the gut mucosa. This step involves *C. difficile* adhesion to the epithelium mucosa and multiplication, with concomitant evasion of the host immune system. *C. difficile* produces several surface proteins that may contribute to colonization and pathogenesis^7,8^. Among the *C. difficile* cell wall proteins (CWPs), the two components of the S-layer (LMW-SLP and HMW-SLP)^9,10^ and the CWP66 protein^11–13^ have been shown to have adhesive properties and to act as colonization factors. Other surface proteins likely involved in the host colonization are the heat shock protein GroEL^14,15^, the flagellar proteins FliC and FliD^16,17^ and the lipoprotein CD0872^18^.

As other pathogens, *C. difficile* targets host molecules via multiple mechanisms, including binding to components of the host extracellular matrix (ECM). The surface proteins known to mediate adhesion to ECM are designated as Microbial Surface Components Recognizing Adhesive Matrix Molecules (MSCRAMMs). Their role could be crucial in *C. difficile* pathogenesis as the disruption of epithelial barriers caused by the toxins determines the accessibility to the underlying matrix and therefore favors bacterial colonization. Fibronectin-binding protein A (FbpA) and Collagen-binding protein A (CbpA) are examples of *C. difficile* MSCRAMMs playing a role in this mechanism^19,20^.

A second role for MSCRAMMs has been identified in the host immune system evasion with the binding to the complement component C1q. The collagen-binding protein Cna from *Staphylococcus aureus* and other similar proteins of Gram-positive bacteria can bind to the C1q “collagenous domain”, a sequence of approximately 81 residues representing the stem of the bouquet-like structure of C1q. Interaction of Cna with C1q has been shown to prevent the binding of C1r to C1q, thus inhibiting the C1 complex formation and consequently the classical complement pathway activation^21,22^.

*C. difficile* colonization is regulated by the second messenger c-di-GMP, whose levels control certain proteins expression through binding to riboswitches located upstream of the target genes^23^. *C. difficile* encodes a large number of diguanylate cyclases and phosphodiesterases that are responsible for the synthesis and degradation of c-di-GMP, and 16 predicted c-di-GMP-responsive riboswitches have been identified in the genome of *C. difficile*^24–26^. C-di-GMP controls bacterial physiology and virulence by modulating the expression levels of *tcdA* and *tcdB*, as well as that of *tcdR*, which encodes an alternative sigma factor that activates *tcdA* and *tcdB* expression^27,28^.

In many bacterial species, c-di-GMP also controls the lifestyle switch from free-living motile state to biofilm communities^29,30^. In *C. difficile*, c-di-GMP negatively regulates flagellar swimming motility and positively regulates expression of putative adhesins and type IV pili (TFP) biosynthesis. Indeed, TFP have been recently shown to be fundamental for adherence and persistence of *C. difficile* in a mouse model of infection^31–34^. C-di-GMP positively regulates *C. difficile* biofilm formation and a high level of intracellular c-di-GMP in the 630 strain results in a more robust biofilm^26,35^. In the biofilm assay, bacteria have been shown to be embedded in a matrix enriched with extracellular DNA, polysaccharides and proteins, including toxin A and B. Besides c-di-GMP, biofilm formation process is modulated by the master regulator Spo0A, as in strain R20291 genetic inactivation of spo0A exhibits decreased biofilm formation^36^. Also, CWP84, flagella, TFP and the quorum-sensing regulator LuxS are required for maximal biofilm formation by *C. difficile*^37–39^. Importantly, evidences support *C. difficile* biofilm formation *in vivo*. In a mouse model, *C. difficile* forms clumps associated with damaged tissue that suggest the formation of microcolonies^40^; infected hamsters and mice have been reported to present aggregation or clusters of *C. difficile*^41,42^ and recent studies showed presence of *C. difficile* biofilm communities in association with the gut mucus layer^42,43^.

In this study we characterized CD2831, a protein with a type II c-di-GMP riboswitch, positively regulated by high levels of intracellular c-di-GMP. Interestingly, a type I riboswitch that is activated at low levels of c-di-GMP, was found in the upstream gene coding for the metalloprotease ZmpI/PPEP-1 (CD2830). CD2831 and another c-di-GMP controlled putative adhesin, CD3246, are recognized and cleaved by ZmpI/PPEP-1. Consistently, it was demonstrated that at low c-di-GMP levels, ZmpI/PPEP-1 is produced and cleaves the low amount of CD2831 and CD3246 present, thus limiting bacterial adhesion to the host^44–46^.

We confirmed that CD2831 is a collagen-binding protein contributing to *C. difficile* adhesion to the host ECM. Overexpression of CD2831 significantly increased biofilm formation *in vitro*, suggesting a role for CD2831 in this process. Moreover, as other MSCRAMMs, CD2831 can bind to human complement C1q and thus potentially be involved in host immune evasion mechanisms.

## Results

### CD2831 domains prediction

**Figure 1:**
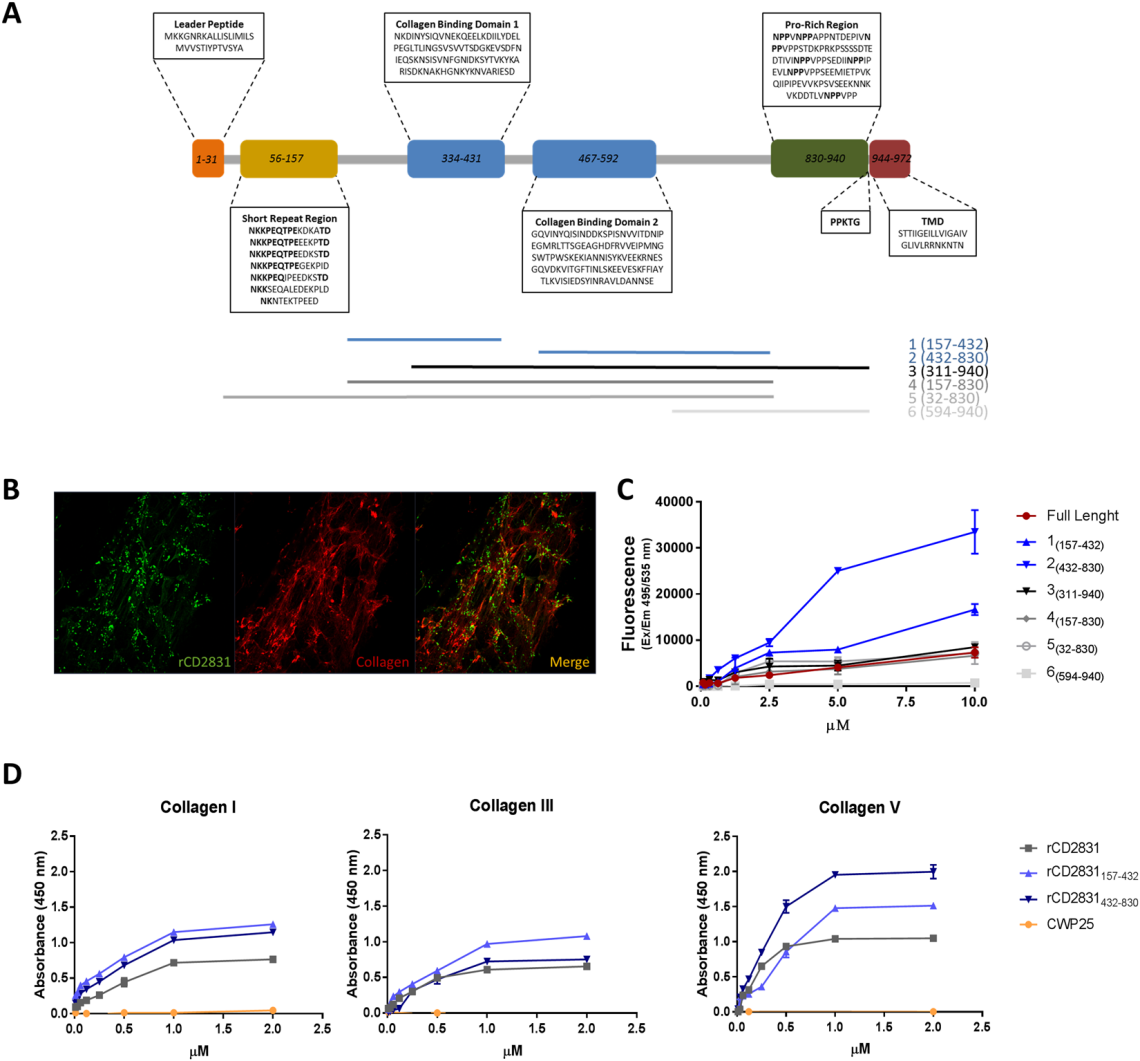
Binding of recombinant CD2831 full-length protein and subdomains to collagen. (**A**) Schematic representation of CD2831 domains organization displays the Leader Peptide at the N-terminus (orange) followed by a Short Repeat Region (yellow) in which repeated amino acids sequences are marked in bold, two collagen-binding domains (blue) and a Proline-rich region (green) recognized by the protease ZmpI/PPEP-1 that cleaves within the seven NPP amino acids sequences of the domain. The charged tail and the hydrophobic domain are present at the C-terminus (red). The protein is cleaved between the threonyl and glycil residues of the PPKTG motif and transferred to the cell-wall peptidoglycan by the Sortase B enzyme. Blue, black and grey lines indicate the length of recombinant CD2831 subdomains compared to the full-length. (**B**) Immunofluorescence of 1 µM of CD2831 (green) binding to collagen (red) produced by IMR90 cells. Collagen staining results from a mix of antibodies against collagen types I, III and V in a 1:1:1 ratio. Binding of the proteins was detected by using rabbit anti-CD2831 serum and secondary Alexa Fluor-conjugated antibodies. (**C**) Human fibroblasts IMR90 were cultured into collagen I-coated 96 well plates for three days after seeding. Cells were then incubated with serially diluted recombinant CD2831 proteins ranging from 0.08 to 10 µM. Binding of CD2831 was detected as above and quantified with a microplate fluorescence reader. (**D**) ELISA plates were coated with 10 μg/ml of purified collagens and incubated with serially diluted recombinant CD2831 proteins ranging from 15 nM to 2 µM. Binding of the proteins was detected using rabbit anti-CD2831 serum, followed by HRP-conjugated-secondary antibody. CWP25 protein was used as negative control. CD2831 CBD1 and CD2831 CBD2 represent the previously described subdomains 1 and 2.

The approximately 108 kDa protein CD2831 presents an N-terminal putative leader peptide of 31 amino acids followed by a short repeat region (AA 56-157). Despite a previous study identified the presence of a central “Collagen-Hug” motif between amino acids L173 and G459^44^, our prediction study revealed the presence of a collagen-binding superfamily region spanning from residues 225 to 797. Weak sequence similarities (20% amino acid identity) to the *S. epidermidis* Adhesin Sdrg and the *S. pyogenes* T6 Backbone Pilin were detectable in the 165–460 and 461–820 regions respectively, suggesting the typical MSCRAMM structural organization in IgG-like sub-domains. Localization of putative collagen-binding domains was further investigated and predicted the location of two collagen-binding domains (CBDs), from here on indicated as CBD 1 and 2. The first extends from amino acids N334 to D431 whereas the second corresponds to the region from G467 to E592. The CBDs are followed by a region of 110 amino acids rich in Proline that has been showed to be targeted by ZmpI/PPEP-1^44,45^. Interestingly, as described above, CD2831 and ZmpI/PPEP-1 are inversely regulated by c-di-GMP through a riboswitches mechanism. The C-terminal region encompasses an LPXTG-like motif (PPKTG) recognized by the Sortase B enzyme, which is responsible for the cell-wall proteins attachment to peptidoglycan^45^ followed by a hydrophobic region, likely representing the transmembrane domain (Fig. 1A).

### Recombinant CD2831 and subdomains containing CBD1 and CBD2 bind to native collagen produced by cells and to collagen types I, III and V

To investigate the predicted collagen-binding activity of CD2831, the sequences coding for the full-length CD2831 and six different portions of the protein were cloned and expressed in *E. coli*. Subdomains 1 (157–432) and 2 (432–830) include the individual CBD1 and CBD2 respectively. CD2831 subdomains 3 (311–940), 4 (157–830) and 5 (32–830) encompass both the CBDs, with subdomain 3 including also the Pro-rich region and subdomain 5 the short repeat region. Subdomain 6 (594–940) represents the C-terminus of CD2831 and, lacking any CBD, was used as negative control in the binding assays (Fig. 1A).

After purification, full-length CD2831 (SDS-PAGE of recombinant soluble protein showed in Fig. S4) and subdomains were tested in their binding activity towards native collagen produced by human cells. For this, confluent IMR90 human fibroblasts were cultured on 96-well plates for three days. In these conditions IMR90 cells produce a considerable amount of ECM, with a relevant presence of collagen. Full-length recombinant CD2831 binding to native collagen was visualized by immunofluorescence, which displays a spatial co-localization between CD2831 (green) and native collagen (red) produced by IMR90 human fibroblasts (Fig. 1B). Experiments using IMR90 showed that CD2831 displays a dose-dependent binding activity to native collagen. Subdomains 3 (311–940), 4 (157–830) and 5 (32–830), encompassing the CBD1 and CBD2 portions, showed a binding kinetic similar to the full-length protein, confirming the predicted collagen binding activity. Addition of the short repeat region at N-terminal (subdomain 5) or the Proline-rich region (subdomain 3) at C-terminal showed no impact on binding activity, further confirming that the minimal domain mediating the interaction to collagen resides in the CBD1 and CBD2 portions of the protein. When expressed individually, CBD1 and CBD2 (subdomains 1 and 2) showed higher binding activity compared to the full-length protein (Fig. 1C).

To further define the affinity of CD2831 to the collagen types known to be more represented in the gut tissue^47^, collagen-binding activity of CD2831 full-length and subdomains 1 and 2 (containing single CBD) was further investigated through ELISA on immobilized collagen type I, III and V. Recombinant proteins in a range of concentrations between 0.15 and 2 µM showed a dose-depending binding activity to collagen types I, III and V. All the recombinant proteins showed a higher binding affinity to collagen type V, whereas the affinity for collagens types I and III were comparable. Consistent with what observed in the experiments described above with human cells, binding of single CBD (subdomains 1 and 2) revealed to be higher compared to the full-length CD2831 (Fig. 1D**)**.

### CD2831 overexpression enhances *C. difficile* binding to immobilized collagen and to native collagen produced by human IMR90 cells

**Figure 2:**
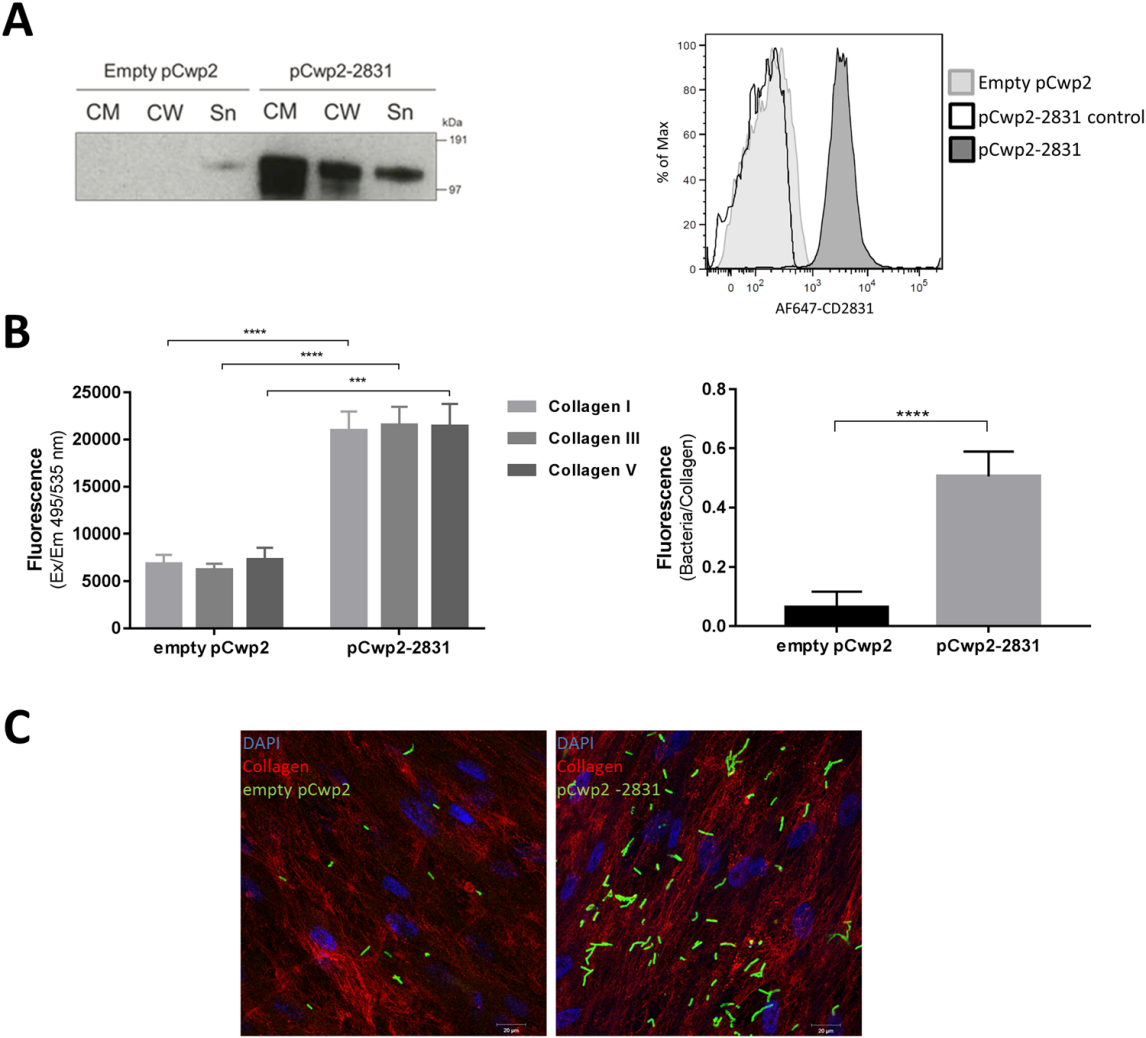
CD2831 overexpression increases *C. difficile* binding to collagens and human fibroblasts. (**A**) (Left panel) Overnight cultures of *C. difficile* overexpressing CD2831 (pCwp2-2831) and control (empty pCwp2 vector) strains were centrifuged and proteins released in the supernatant (Sn) were TCA precipitated. The cell wall (CW) was isolated from cytoplasm and membranes (CM) and the three fractions were analyzed by western blot using rabbit anti-CD2831 serum. Full-length western blot showed in Figure S1. (Right panel) Flow-cytometry analysis of CD2831 surface exposure: in light grey is showed the wild type condition (empty pCwp2 vector), in dark gray is the overexpressing condition (pCwp2-2831). The negative black line represents the overexpressing strain stained only with the secondary antibody as negative control. (**B**) Adhesion of *C. difficile* overexpressing CD2831 and control strains to immobilized collagens type I, III and V (left panel) and to IMR90 cells (right panel) after 1-hour incubation in anaerobic condition. Adhering bacteria were stained by using anti-CD630Δ*erm* serum followed by Alexa-fluor conjugated secondary antibody and revealed by a fluorescence plate reader. Adhesion of *C. difficile* to cells was normalized for the levels of collagens produced by cells in each well. (**C**). Confocal images of *C. difficile* control and CD2831-overexpressing strains adhering to the collagen-producing IMR90 cells, after 1-hour incubation at 37 °C in anaerobic conditions. Cells nuclei are stained with DAPI (blue), bacteria are showed in green and collagen in red.

CD2831 is poorly detected in both the supernatant and the whole cell lysate of strain 630Δ*erm* due to the very low levels of c-di-GMP in *in vitro* conditions. Stimuli inducing high levels of c-di-GMP *in vitro* are unknown, therefore to investigate the activity of CD2831 in the context of the bacterial cell wall, the gene was constitutively overexpressed in *C. difficile* under the control of the *cwp2* promoter. The wild type strain containing only the empty vector presented CD2831 exclusively in the supernatant (Sn), whereas in the overexpressing strain (pCwp2–2831), CD2831 was also detected in the fraction containing cytosol and membranes (CM) and was associated with the cell wall (CW). Cell wall expression of CD2831 was also investigated and confirmed by flow-cytometry (Fig. 2A).

The adhesive properties of CD2831 were explored by analyzing *C. difficile* 630Δ*erm* constitutively overexpressing CD2831 (pCwp2–2831) in binding to immobilized collagen types I, III and V in compared to controls. Cultures of *C. difficile* strains grown at late-exponential phase (OD_600_ 0.8–1) were inoculated into collagen-coated plates and bacteria were incubated for one hour. These experiments showed that the overexpression of CD2831 confers significantly increased adhesiveness of *C. difficile* to immobilized collagens, compared to the control strain (Fig. 2B). Likewise, we also measured the adhesion of CD2831-overexpressing *C. difficile* to collagen-producing cells IMR90. Quantification of fluorescent adhering-bacteria and confocal microscopy analysis showed that CD2831 overexpression determined a significant increase of *C. difficile* adhesion to native collagen-producing cells (Fig. 2B,C).

### Contribution of CD2831 to biofilm formation

**Figure 3:**
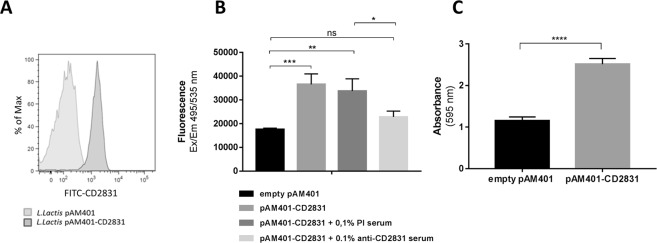
CD2831 contribution to biofilm formation. (**A**) Flow-cytometry analysis of CD2831 expressed in *C. difficile* 630Δ*erm*. Bacteria were cultured either in BHIS for 3 hours (light grey peak) or in BHIS + 2% (w/v) glucose on plastic plates for 3 days for biofilm formation (dark grey peak). (**B**) Overnight cultures of *C. difficile* constitutively expressing CD2831 and control strains were diluted in BHIS + 2% (w/v) glucose and allowed to form biofilm for 24 hours. Bacterial biofilm was stained with crystal violet, methanol-extracted dye was diluted 1:10 and absorbance was measured at 595 nm (left). Z-stack confocal microscopy of 3-day biofilm from *C. difficile* bacteria. Bacterial nuclei and extracellular DNA were stained using DAPI (right). (**C**) Western blot showing CD2831 expression in bacterial lysates and supernatants of *C. difficile* pTet-CD2831 after induction with 0, 25, 50 and 100 ng/mL of anhydrotetracycline (ATc) for 24 hours (left). Full-length western blot showed in Figure S2. *C. difficile* pTet-CD2831 strain 24-hour biofilm was measured by crystal violet staining after inducing CD2831 expression, using different concentration of ATc. Methanol-extracted dye was diluted 1:2 (right).

The second messenger c-di-GMP has been shown to be involved in controlling *C. difficile* lifestyle switch from motility to sessility. Specifically, this results in the down-regulation of flagella and toxins in favor of an increased expression of adhesins or biofilm-related proteins. As a c-di-GMP regulated adhesin, we also investigated CD2831 for playing a role in biofilm formation. To check if CD2831 is expressed during biofilm formation, *C. difficile* was allowed to grow in biofilm on abiotic surface for three days and after removal of the planktonic bacteria, biofilm was detached, washed and analyzed by flow-cytometry. When compared to exponentially-growing bacteria, biofilm-detached bacteria showed higher levels of CD2831 on the cell wall suggesting enhanced expression of the cell wall-associated protein during the biofilm phase (Fig. 3A**)**.

*C. difficile* overexpressing CD2831 (pCwp2–2831) was also compared with a control strain carrying the empty vector, in the ability to form biofilm on an abiotic surface. The crystal violet assay showed a two-fold increase in bacterial biomass formation in the CD2831-overexpressing strain compared to the control, after 24 hours (Fig. 3B, left panel). To measure biofilm thickness by confocal microscopy, an increased bacterial biomass was required so *C. difficile* carrying pCwp2-2831 or the empty vector were incubated for three days on glass chamber slides. Z-stack confocal microscopy images confirmed a two-fold increase in thickness of bacterial biofilm (Fig. 3B, right panel). To modulate CD2831 expression levels in *C. difficile* more precisely, plasmid pTet-2831, carrying CD2831 under the control of the pTet-inducible promoter, was constructed and introduced into the wild-type 630Δ*erm* strain. Expression was then induced by a range of anhydrotetracycline (ATc) concentrations, and CD2831 levels were determined by immunoblotting. pTet promoter controlling CD2831 was induced with ATc (0, 25, 50 and 100 ng/ml) and biofilm formation was measured after 24-hour growth on a plastic surface. The biofilm was then measured through crystal violet assay that clearly showed that *C. difficile* biofilm increased proportionally to the expression of CD2831 (Fig. 3C).

### Heterologous expression in *Lactococcus lactis* surface confirmed role of CD2831 in adhesion to collagen and biofilm formation

**Figure 4:**
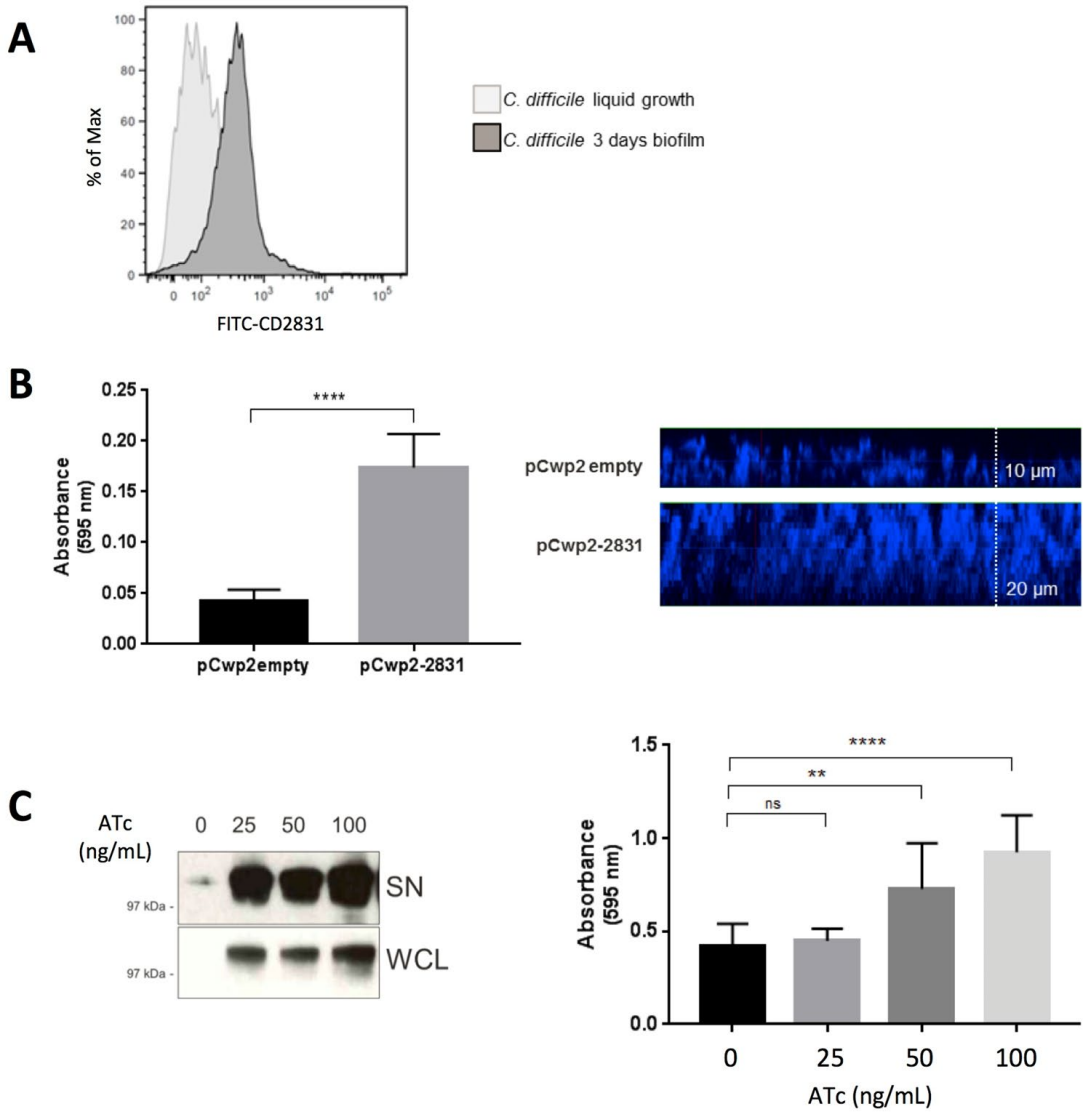
*L. lactis* as heterologous system to study CD2831 adhesive properties. (**A**) Flow**-**cytometry analysis showing surface exposure of CD2831 in *L. lactis* transformed with pAM401-CD2831. (**B**) *L. lactis* strains were allowed to adhere to ECM-producing IMR90 human fibroblasts for 1 hour at 37 °C. Data show the contribution of CD2831 in adhesion of *L. lactis* to cells and inhibition of *L. lactis* expressing CD2831 adhesion upon pre-incubation of bacteria with 0.1% (v/v) rabbit anti-CD2831 serum for 1 hour at 37 °C. (**C**) *L. lactis* strains were cultured as biofilm on abiotic surface for 24 hours. Bacterial biomass was measured by crystal violet, methanol-extracted dye was diluted 1:2 and absorbance at 595 nm was measured.

A useful tool to demonstrate putative adhesive properties of proteins of interest is the heterologous expression on the surface of *Lactococcus lactis*^20,48–50^. We constitutively expressed CD2831 in *L. lactis* surface using the pAM401 vector and the contribution of the heterologous protein expression to adhesion was evaluated. Protein anchoring to *L. lactis* cells was achieved by substituting the native cell wall anchoring motif of CD2831 with a previously described motif^51^. Flow-cytometry analysis was used to demonstrate that export of CD2831 on *L. lactis* surface was successfully obtained (Fig. 4A). This allowed us to generate a strain in which CD2831 was exclusively cell wall-associated. *L. lactis* expressing CD2831 was investigated for its binding activity to collagen-producing cells and, as observed for *C. difficile*, expression of CD2831 conferred an advantage in binding to IMR90 human fibroblasts. Moreover, a pre-incubation of *L. lactis* with 0,1% CD2831 anti-sera was sufficient to reduce CD2831-expressing *L. lactis*- adhesion to IMR90 reaching the same level as wild type *L. lactis* (Fig. 4B).

The *L. lactis* tool was employed to study the contribution of CD2831 in bacterial biofilm formation. As for *C. difficile*, *L. lactis* expressing CD2831 and control strains were allowed to grow in biofilms on an abiotic surface for 24 hours. Crystal violet assays showed that also in the *L. lactis* bacterial background, the overexpression of CD2831 contributes to increased biofilm formation (Fig. 4C).

### CD2831 binds to human complement C1q

**Figure 5:**
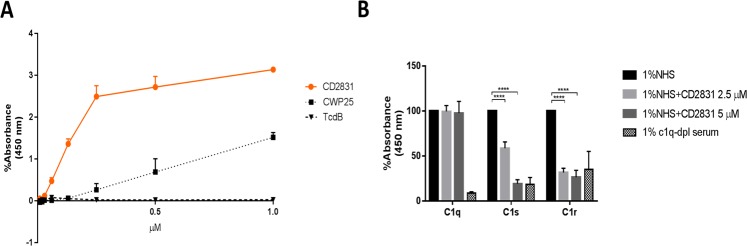
Recombinant CD2831 binding to human complement C1q. (**A**) ELISA plates coated with 10 µg/ml of purified human complement component C1q were incubated with several dilutions of recombinant CD2831 (15 nM-1 µM) for 1 hour. *C. difficile* recombinant TcdB and CWP25 were used as control. (**B**) ELISA plates coated with 10 µg/ml purified human IgM were incubated with 1% (v/v) Normal Human Serum (NHS) and different concentrations of recombinant CD2831. After 1 hour, deposition of human C1q, C1s and C1r was measured. 1% (v/v) C1q-depleted serum (C1q-dpl) was used as negative control.

In addition to binding to matrix components, proteins with collagen-binding domains from Gram-positive bacteria are also known to bind to human complement C1q and to act as inhibitors of the classical complement activation pathway^21^. To test this, we analyzed recombinant full-length CD2831 binding to human C1q. ELISA on immobilized C1q was performed and a dose-range binding activity of CD2831 towards C1q was registered (Fig. 5A) with CWP25, the more abundant protein in the cell wall architecture of the S-layer^52^, included as control. The CD2831-C1q interaction was also investigated by Transmission Electron Microscopy (TEM). TEM images showed that CD2831 displays a “comma-like” shape and notably, when incubated in a 1:1 molar ratio with human C1q, binding of CD2831 to the C1q stalk (also known as “collagen-like” region) was detected (Fig. S3).

To initiate this immune defense mechanism, the C1q stalk must be recognized by two other molecules: C1s and C1r. Together these proteins interact and form the C1 complex, which activates the classical pathway of the complement cascade. To investigate interference of CD2831 in C1 complex formation, we performed ELISA on immobilized IgM in which we measured C1q, C1s and C1r deposition in presence of different concentrations of recombinant CD2831. ELISA results showed that recombinant CD2831 binding to C1q stalk does not have an impact on C1q recognition of Fc regions of IgM, but reduces C1s and C1r association with C1q, thereby compromising C1 complex formation (Fig. 5B).

## Discussion

Bacterial infections involve binding of pathogens to ligands expressed on the surface of host cells and in the ECM^53–55^. In an intact healthy tissue, ECM is not exposed to the environment and may not be accessible for interaction with pathogens. A peculiar feature of *C. difficile* pathogenesis is the disruption of the intestinal epithelial tight junctions caused by the glycosylating toxins TcdA and TcdB resulting in consequent exposure of the underlying ECM^56^. In these conditions, bacterial matrix-binding proteins can recognize ECM ligands, such as fibronectin or collagen and thus operate as virulence factors. To date, high-molecular weight S-layer protein (HMW-SLP) and Collagen-binding protein A (CbpA) of *C. difficile* have been described as surface proteins that are able to bind to collagen and that could be implicated in this mechanism^10,20^. CD2831 is another collagen-binding protein of *C. difficile* whose presence on the bacterial surface is tightly regulated by the endogenous protease ZmpI/PPEP-1, that is abundant in low intracellular c-di-GMP levels conditions and efficiently cleaves CD2831, allowing its release in the extracellular milieu.

Hensbergen *et al*. showed that recombinant CD2831 collagen hug domain (CHD) was able to recognize collagen type I spotted on polyvinylidenefluoride membrane strips^44^. We analyzed the CD2831 protein sequence and identified two predicted collagen-binding domains, CBD1 and CBD2. We then cloned and expressed the full-length CD2831, the individual CBD1 and CBD2 domains and three other subdomains containing both CBDs in combination with additional portions of the protein. Collagen-binding assays confirmed CD2831 as a protein able to bind to collagen types I, III and V and to native collagen produced by human cells. Similar results were obtained for subdomains containing both CBDs confirming that the minimal portion mediating the interaction to collagen resides in the two predicted CBDs. In these experiments, the recombinant proteins presenting a single CBD, showed higher affinity to collagen compared to the full-length CD2831. This result could be attributed to better exposure of the CBD to its ligand when the domain itself is expressed singly. However, we cannot exclude that the full-length protein is incorrectly folded when expressed in *E. coli* or that its folding partially occludes the CBDs. In absence of the native protein purified from *C. difficile* both hypothesis are possible. When grown *in vitro*, the level of CD2831 associated to the bacterial cell wall^44,45^ is extremely low, as a consequence of almost undetectable levels of *C. difficile* c-di-GMP. Physiological stimuli increasing c-di-GMP levels are still unknown but it is clear that it coincides with the switch of *C. difficile* to a sessile lifestyle, as c-di-GMP positively regulates adhesins such as TFP and negatively controls flagellar motility. Presence of bile salts as a signal upstream c-di-GMP regulation has been described for *Vibrio cholerae*^57^. Nevertheless, incubation of *C. difficile* with bile acids mixtures did not drive c-di-GMP increase in our experimental conditions (data not shown). Overexpression of diguanylate cyclases and genetic depletion of phosphodiesterases are the approaches followed in several studies for increasing c-di-GMP levels in *C. difficile in vitro*. However, in these conditions, the bacterial cell wall is dramatically changed by the effects of c-di-GMP on many proteins and may not be the optimal assay for studying single protein functions. Our approach was to overexpress CD2831 in *C. difficile* and, although a considerable amount of the protein was still cleaved by ZmpI/PPEP-1, a part was clearly cell-wall associated and able to promote the collagen-binding activity toward immobilized collagens and native collagen produced by human cells. Contrarily to the results presented here, overexpression of CD2831 in previous studies showed no detectable proteins associated to the cell wall^45^. These discrepancies could be due to the differences in the bacterial growth conditions or in the levels of Sortase B and protease PPEP-1 expression.

To overcome protease cleavage and release of CD2831 by ZmpI/PPEP-1, which targets also the other putative adhesin CD3246^45^, we employed heterologous expression of CD2831 in *L. lactis* and confirmed collagen binding activity of CD2831 also in this bacterial background.

Taken together, these results demonstrate that CD2831 is a member of the MSCRAMMs family. Other MSCRAMMs of Gram-positive bacteria, such as the fibronectin-binding protein FbsC of *Group B streptococcus*, have been shown to be involved in host epithelium invasion and in *in vitro* biofilm formation^58^. This, together with positive regulation of CD2831 mediated by c-di-GMP (which also positively modulates *C. difficile* biofilm) led us to think that CD2831 could be involved in biofilm formation. After confirming increased levels of CD2831 on *C. difficile* during biofilm formation, we compared CD2831-overexpressing *C. difficile* with the control strain in their ability to form biofilm *in vitro* and we showed that overexpression of CD2831 raised bacterial biomass formation. Also, we were able to observe the same phenotype in CD2831-expressing *L. lactis*. This could be reasonably explained as result of an increased aggregative phenotype in bacteria overexpressing CD2831. Recently, Poquet and co-workers showed that *C. difficile* biofilm was not affected by the absence of CD2831 in a clostron knock-out mutant, concluding that this protein is dispensable for biofilm formation^59^. However, the lack of CD2831 could be still compensated by other biofilm-related proteins, masking the effect of CD2831 deficiency. Of course, we are not able to establish to what extent CD2831 contributes to biofilm formation, due to the absence in our experiments of a positive control represented by a strain overexpressing type IV pili or other proteins known to positively regulate biofilm formation in *C. difficile*.

Kang and co-authors recently showed that the MSCRAMM Cna protein from *Staphylococcus aureus* can interfere with the complement cascade activation by interacting with human complement C1q^21^. Host adaptive immune response against bacteria starts with C1q binding to the Fc region of antibodies surrounding the bacterial surface. This initiates the complement cascade via the classical pathway that proceeds with the C3b opsonization of the pathogen and the associated enhanced phagocytosis. Complement cascade also leads to the generation of chemotaxis factors such as C3a and C5a involved in phagocytes recruitment. To initiate this immune defense mechanism, the C1q stalk must be recognized by two other molecules called C1r and C1s. Association of these proteins leads to the formation the C1 complex, which activates the classical pathway of the complement cascade^60^. Cna-C1q interaction was shown to inhibit complement activation via the classical pathway^21^. Considering this dual role of Cna and other MSCRAMM proteins, we investigated CD2831 binding to C1q. Besides confirming a dose-range affinity of CD2831 to human C1q, we also visualized the formed complex by transmission electron microscopy (TEM), which showed CD2831 interacting with the collagen-like domain of the C1q protein stalk, corresponding to the region where the C1r and C1s associate to form the heterotetramer (C1r2C1s2). We performed ELISA to measure C1q, C1s and C1r deposition in presence of different concentration of recombinant CD2831. Whereas C1q recognition of IgM is not altered by the presence of CD2831, we were able to demonstrate that recombinant CD2831 binding to C1q stalk reduced C1s and C1r deposition on IgM-associated C1q. In the presence of CD2831, the C1 complex formation is therefore abolished and the complement cascade cannot be activated via the classical pathway.

Importantly, in the late 1980s, a role for C1q independent from complement activation emerged with the observation that immobilized C1q, in the absence of C1r and C1s, triggered an enhancement of FcγR-mediated phagocytosis when targets were coated with a sub-optimal concentration of antibody^61^. Moreover, this activity required the collagen-like tail of C1q and extended to enhancement of CR1-mediated phagocytosis^62^. One of the primary features of *C. difficile* colitis in humans is the presence of an inflammatory infiltrate in the colonic mucosa characterized by epithelial cell necrosis and presence of neutrophils in the colonic lumen. Experiments in intact animals demonstrated that injection of toxin A into ileal or colonic loops stimulates secretion of fluid accompanied by increased mucosal permeability, epithelial cell destruction, and infiltration of neutrophils^63^, which are potentially able to contrast the infection. Due to its main functions in the opsonization and elimination of bacteria, the complement system is crucial for the efficient clearance of pathogens and, although the complement-mediated response against *C. difficile* in the gut infection remains elusive, we are led to believe that this bacterium does not represents an exception to these mechanisms. All together, these observations suggest that microbial MSCRAMMs binding to C1q is beneficial for pathogens even during early stages of infection, when production of anti-microbial antibodies is still not efficient, and the employment of this mechanism could be crucial for bacteria to evade recognition by macrophages residing in the gut mucosa^64^.

In conclusion, we have characterized the novel collagen-binding protein CD2831 that contributes to host colonization and biofilm formation. These functions of CD2831 are most likely mediated by the cell wall-associated form of the protein and this was further confirmed in the *L. lactis* model, in which the specific ZmpI/PPEP-1 protease is not present. On the other hand, because of its complement inhibition activity, CD2831 represents for *C. difficile* a defense weapon against the immune system and we can not exclude that binding to C1q could be mediated by the ZmpI/PPEP-1-cleaved soluble form of CD2831.

## Methods

### Bacterial strains and growth conditions

*C. difficile* strain 630Δ*erm* was grown anaerobically at 37 °C in BHIS (ThermoFisher, USA). *E. coli* HK100 cells and BL21(DE3)T1^R^ strains, were cultured at 37 °C in Luria-Bertani (LB) + 100 µg/ml ampicillin. *L. lactis* MG1363 strain was cultured at 30 °C in M17 medium + 0.5% glucose and, when required, with 20 µg/ml chloramphenicol. Anhydrotetracycline (aTC) was used (10–100 ng/ml) for induction of the pTet promoter in *C. difficile*.

### Cloning, expression and purification of CD2831

CD2831 domain prediction was assessed with a blast search against the CDD database available at NCBI^65^. Localization of collagen-binding domains was further investigated with Pfam (https://pfam.xfam.org/). For the expression of recombinant CD2831, the corresponding full-length sequences (aa 32–940) was amplified by PCR using chromosomal DNA from *C. difficile* as template (primers listed in table S1) and cloned into pET21 vector (Novagen, USA) using the PIPE method^66^. Codon-optimized synthetic genes corresponding to domains 1–6 were purchased from GeneArt Gene Synthesis (ThermoFisher, USA) and cloned in pET21. Protein expression was induced using 0.5 mM Isopropyl β-D-thiogalactopyranoside (IPTG). Recombinant CD2831 His-tagged proteins were purified by affinity chromatography.

### Construction of CD2831 overexpressing strains of *C. difficile*

The CD2831 gene was amplified from *C. difficile* genomic DNA. The region containing the ribosome binding site (RBS) of *C. difficile* SlpA protein was included in the forward primer (Table S1). The PCR product was cloned into pMTL960 derivative vectors^67^ containing either the *cwp2* constitutive promoter (producing pCwp2-2831) or the pTet inducible promoter (producing pTet-2831).

### *E. coli-C. difficile* conjugation

Overnight cultures of *E. coli* SM10λpir carrying pCwp2/pTet-CD2831 were harvested and resuspended in 200 μl of *C. difficile* overnight culture. The bacterial suspension was spotted onto BHIS agar plates and conjugation proceeded for 8 hours. 1:5 dilutions were plated onto BHIS agar containing 20 μg/ml thiamphenicol for plasmid selection and 250 μg/ml cycloserine for *E. coli* growth inhibition. Plates were grown overnight at 37 °C anaerobically. Transconjugants were picked and streaked twice onto fresh selective plates.

### Generation of *Lactococcus lactis* expressing CD2831 strain

The construction of *L. lactis* expressing CD2831 was performed as previously described^20^. Briefly, the CD2831 gene was amplified by PCR using chromosomal DNA from *C. difficile* as template (Table S1) and cloned into pAM401 expression vector^48^. The coding sequence for the leader peptide (1–93 bp) and the PPKTG-motif at the C-terminus (2817–2829 bp) of CD2831 were substituted with the same regions of the GAS *emm1* gene encoding the streptococcal M1 protein to ensure the surface exposure in *L. lactis* as described by Edwards *et al*.^51^. pAM401-M1 was used as template to amplify the expression vector containing the leader peptide and the C-terminal of M1 protein (Table S1). The two products were assembled by the PIPE method in *E. coli* HK100 cells and the resulting vector was introduced in *L. lactis* MG1363 by electroporation.

### Production of polyclonal antibody against CD2831

Rabbits were immunized three times, 2 weeks apart, with 10 μg of recombinant CD2831 in the presence of aluminum hydroxide. The immune serum was obtained 14 days after the last immunization. All animal experiments were performed in accordance with relevant guidelines and regulations established by the Italian law, including the approval of the local Animal Welfare Body followed by authorization of Italian Ministry of Health.

### Cell fractionation and protein analysis

To obtain *C. difficile* cell wall-associated proteins, bacteria were grown until exponential phase, harvested by centrifugation and washed in Tris-Sucrose buffer (TS: 10 mM Tris-HCl pH 6.9, 10 mM MgCl_2_, 0.5 M sucrose). Cells were then resuspended in TS buffer, protease inhibitors cocktail (Complete Mini EDTA-free) and 250 μg/ml of mutanolysin (Sigma, USA) and digestion was allowed to proceed for 2 hours at 37 °C with gentle agitation. Protoplasts were removed by centrifugation at 3000 *xg* for 30 minutes. The supernatant was then subjected to microcentrifugation at 17000 *xg* for 30 minutes and the solubilized cell surface proteins were recovered. Proteins were separated by SDS-PAGE electrophoresis using the NuPage Gel System (ThermoFisher, USA) and transferred to nitrocellulose membranes for Western blot analysis. After blocking with PBS containing 0.05% Tween-20 (PBS-T) and 10% (w/v) milk (Sigma, USA), proteins were detected with rabbit anti-CD2831 serum diluted 1:1000 for 1 hour at RT, followed by the HRP-conjugated secondary antibodies (Dako, Denmark) diluted 1:2000 for 1 hour at RT. Antibodies were diluted in 0.05% PBS-T and 3% (w/v) milk. Bands were visualized with Super Signal® West Pico Chemiluminescent Substrate (ThermoFisher, USA).

### Flow cytometry analysis

*C. difficile* and *L. lactis* cultures were harvested by centrifugation, washed with PBS and resuspended in 4% (v/v) formaldehyde (ThermoFisher, USA) diluted in H_2_O for 30 minutes at RT. After two washes with PBS, bacterial pellets were incubated with rabbit anti-CD2831 serum diluted 1:100 for 1 hour at RT. Following PBS washes, bacteria were incubated with Alexa-Fluor 647-conjugated anti-rabbit IgG secondary antibody (ThermoFisher, USA) diluted 1:500 for 30 minutes at RT. All washing steps and antibodies dilutions were performed using 1% (w/v) bovine serum albumin (BSA) in PBS. Bacterial samples were analyzed by BD FACS Canto II system (BD Bioscience, USA).

### Proteins ELISA

Nunc MaxiSorp PS Immuno F96-plates (ThermoFisher, USA) were coated overnight at 4 °C with 100 μl of collagen types I, III, V or human C1q (Sigma, USA) at the final concentration of 10 μg/ml. Plates were washed three times with 0.9% NaCl + 0.05% Tween-20 (NaCl-T) and blocked with 3% (w/v) BSA + 0.05% PBS-T for 2 hours at 37 °C. Recombinant *C. difficile* proteins were two-fold serially diluted in PBS, then added to the collagen/C1q-coated wells and incubated for 1 hour at 37 °C. Following incubation, the wells were gently washed with NaCl-T. Anti-CD2831 antibodies (1:500 in PBS/BSA) were added to each well and incubated for 1 hour. Wells were washed with NaCl-T before adding HRP-conjugated anti-rabbit IgG secondary antibody (Dako, Denmark), 1:2000 in PBS/BSA for 1 hour. OPD substrate (5 ml 0.1 M citric acid, 5 ml 0.2 M Na_2_HPO_4_, 10 ml H_2_O, 1 OPD tablet (Sigma, USA), 8 μl H_2_O_2_) was added to each well and the reaction was stopped with 3 M H_2_SO_4._ Optical density at 450 nm was determined using a microplate reader.

The competition assay was the previously described ELISA method performed with the following modifications: two-fold serially diluted CD2831 protein was pre-incubated with 1% Normal Human Serum (NHS) (Sigma, USA) at RT for 1 hour. The mixture was added to microtiter plates previously coated overnight at 4 °C with purified IgM (1 μg/well). Rabbit antibodies against C1q, C1s and C1r (Sigma, USA) diluted 1:250 in PBS/BSA were used to detect protein deposition. C1q-depleted serum (Sigma, USA) was used as negative control.

### Proteins binding and bacterial adhesion assays to IMR90 cells

IMR90 human fibroblasts were cultured in Eagle’s Minimum Essential Medium (EMEM) (ATCC, USA) supplemented with 10% heat-inactivated fetal bovine serum (FBS) (ThermoFisher, USA) and antibiotics at 37 °C with 5% CO_2_. Cells were seeded on wells coated with collagen type I (BD BioCoat, UK) and cultured for 3 days. Cells were then incubated with serially diluted recombinant CD2831 proteins (0.08–10 µM). After washing to remove the unbound proteins, cells were fixed with 4% (v/v) formaldehyde in H_2_O for 30 minutes at RT. Samples were washed and incubated with rabbit anti-CD2831 sera (1:500 in PBS/BSA) for 1 hour at RT. After multiple washes, samples were incubated with FITC-conjugated goat anti‐rabbit IgG (1:500 in PBS/BSA) for 1 hour at RT. Binding of the proteins was quantified with a microplate fluorescence reader.

Similarly, for bacterial adhesion assay, *L. lactis* or *C. difficile* strains in late-exponential phase were resuspended in EMEM medium adjusted to OD_600_ 0.2 and added either to wells previously coated with 10 µg/mL of collagen I, III or V or to the cells. After 2 hours of infection in anaerobic conditions, plates were fixed and immunofluorescence staining was performed as follows. To detect collagen-adhering bacteria, an anti-*C. difficile* polyclonal serum (1:500) and a mix of anti-collagen I, III and V (Santa Cruz Biotechnology, USA) (1:250) in PBS/BSA were added for 1 hour, followed by washes and incubation with Alexa-Fluor-conjugated secondary antibodies (1:500) for 30 minutes. Adhering bacteria were measured by using a fluorescence plate reader and normalized to the amount of collagen detected in each well.

For immunofluorescence microscopy, 1 µM of recombinant CD2831 full-length protein was incubated with IMR90 cells for 1 hour at 37 °C. For microscopy visualization of bacterial adhesion to IMR90, wells were incubated with Alexa-Fluor-conjugated phalloidin (ThermoFisher, USA) and DAPI (Sigma, USA). Glass coverslips were mounted with ProLong® Gold antifade reagent (ThermoFisher, USA) and the slides were analyzed with a Zeiss Observer LSM710 confocal microscope.

### Biofilm formation assay

Overnight bacterial cultures were diluted into fresh medium containing 0.1 M glucose on polystyrene plates for the desired time. Measurement of biofilm was assessed with crystal violet (Sigma, USA) as previously described^39,68^. After the required incubation, wells were washed with PBS and incubated with 0.2% (w/v) crystal violet for 30 minutes. Crystal violet was extracted by addition of methanol for 30 minutes and diluted 1:2 or 1:10 in water. Absorbance at 570 nm was measured using a microplate reader.

### Confocal microscopy analysis of biofilm formation

*C. difficile* was grown on glass chamber slides using BHIS + 0.1 M glucose, at 37 °C in anaerobic conditions for 72 hours. After PBS washing, samples were fixed with 4% (v/v) formaldehyde. Extracellular DNA and nuclei were stained by using DAPI. Wells were washed twice with PBS and coverslips were mounted with ProLong Gold Antifade Reagent. Slides were analyzed with a Zeiss Observer LSM710 microscope.

### Statistical analysis

At least three independent experiments, run under the same conditions, were performed for all studies. Results were analyzed with GraphPad Prism Software by applying the Student *t* test for unpaired data. P ≤ 0.05 was considered statistically significant (*P ≤ 0.05; **P ≤ 0.01; ***P ≤ 0.001, ****P ≤ 0.0001).

## Supplementary information

Supplementary
