# Supplementary material for: Dual role of the colonization factor CD2831 in Clostridium difficile pathogenesis
Source: Sci Rep. 2019 Apr 3;9:5554. doi: 10.1038/s41598-019-42000-8 (PMC6447587; doi:10.1038/s41598-019-42000-8)
Supplement: Supplementary file 1 — Supplementary [file 41598_2019_42000_MOESM1_ESM.pdf]

Dual role of the colonization factor CD2831 in *Clostridium difficile* pathogenesis

Vanessa Arato, Gianmarco Gasperini, Fabiola Giusti, Ilaria Ferlenghi, Maria Scarselli, Rosanna Leuzzi

SUPPLEMENTARY

Table S1. Oligonucleotides used for the present study.

Primer	Sequence (5'-3')*	Characteristics or use
pET21a Fw	CTCGAGCACCACCACCAC	Amplification of pET21a vector for PIPE cloning of CD2831 full-length
pET21a Rv	CATATGTATATCTCCTTCTTAAAGT	
CD2831 FL Fw	ggagatatacatatgTCAGAATTAGGAGAGAATAGTCAGA	Amplification of CD2831 full-length for protein expression and purification in *E. coli*
CD2831 FL Rv	gtggtggtgctcgagTGGTGGTACTGGTGGATTTACC	
pAM401-M1 Fw	GATAGTAATGTAGGTCAATTACCATCAACAGGTGAAACAGCTAACCCA	Amplification of pAM401-M1 vector for pipe cloning of CD2831 full-length
pAM401-M1 Rv	GTATCTGCGAAAGTATTTGAACCGTTAGCCTTAACCTCTGTTTGATTCGC	
CD2831-pAM Fw	GAGGTTAAGGCTAACGGTTCAGAATTAGGAGAGAATAGTCAGATTCAAAG	Amplification of CD2831 full-length for protein expression in *L. lactis*.
CD2831-pAM Rv	ACCTGTTGATGGTAATACTGGTGGATTTACCAAAGTATCATCTTTAAC	
CD2831-pMTL Fw	CGAGCTCAATATAATGTTGGGAGGAATTTAAGAAATGAAGAAAGGAAATAGAAAGGCA	Amplification of CD2831 for cloning into pMTL960-derived vectors and protein expression in *C. difficile*
CD2831-pMTL Rv	GACTAGTCTAATTTGTATTTTTATTTCTTCT	

*Underlined bases indicate engineered restriction sites, low-case bases indicate overlapping sequence.

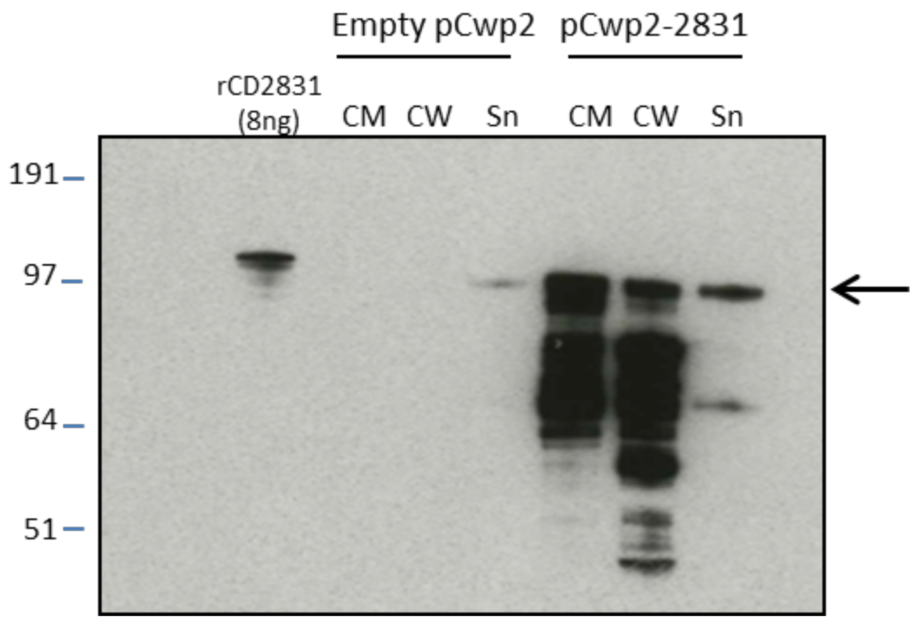

Fig. S1 Constitutive CD2831 overexpression in *C. difficile*

Over-night cultures of *C. difficile* overexpressing CD2831 (pCwp2-2831) and control (pCwp2 empty vector) strains were centrifuged and proteins released in the supernatant (Sn) were TCA precipitated. The cell wall (CW) was isolated from cytoplasm and membranes (CM) and the three fractions were analyzed by western blot using sera raised against CD2831. Bands at 108 kDa indicated by the black arrow represent full-length CD2831, whereas lower bands result from CD2831 degradation observable when the protein is overexpressed and presumably depending to samples preparation. 8 ng of recombinant His-tagged CD2831 was used as control.

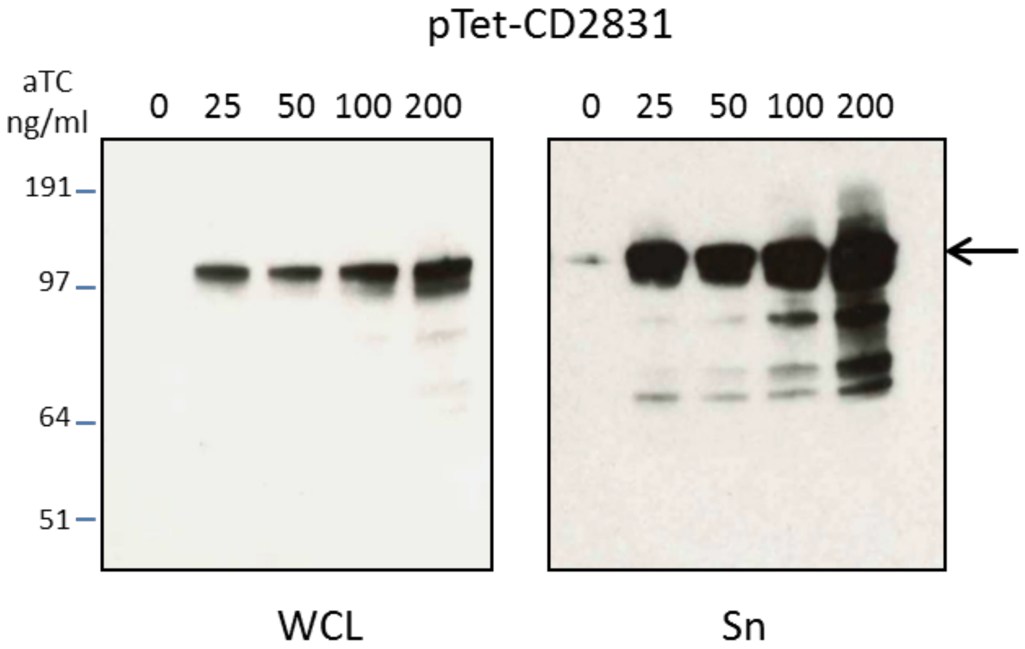

Fig. S2 Inducible CD2831 overexpression in *C. difficile*.

Full-length western blot showing CD2831 expression in bacterial lysates and supernatants of *C. difficile* pTet-CD2831 after induction with 0, 25, 50, 100 and 200 ng/mL of anhydrotetracycline (aTC) for 24 hours. Bands at 108 kDa indicated by the black arrow represent full-length CD2831, whereas lower bands result from CD2831 degradation observable when the protein is overexpressed and presumably depending to samples preparation. Western blots brightness is due to lower exposure of the X-ray films to the blots.

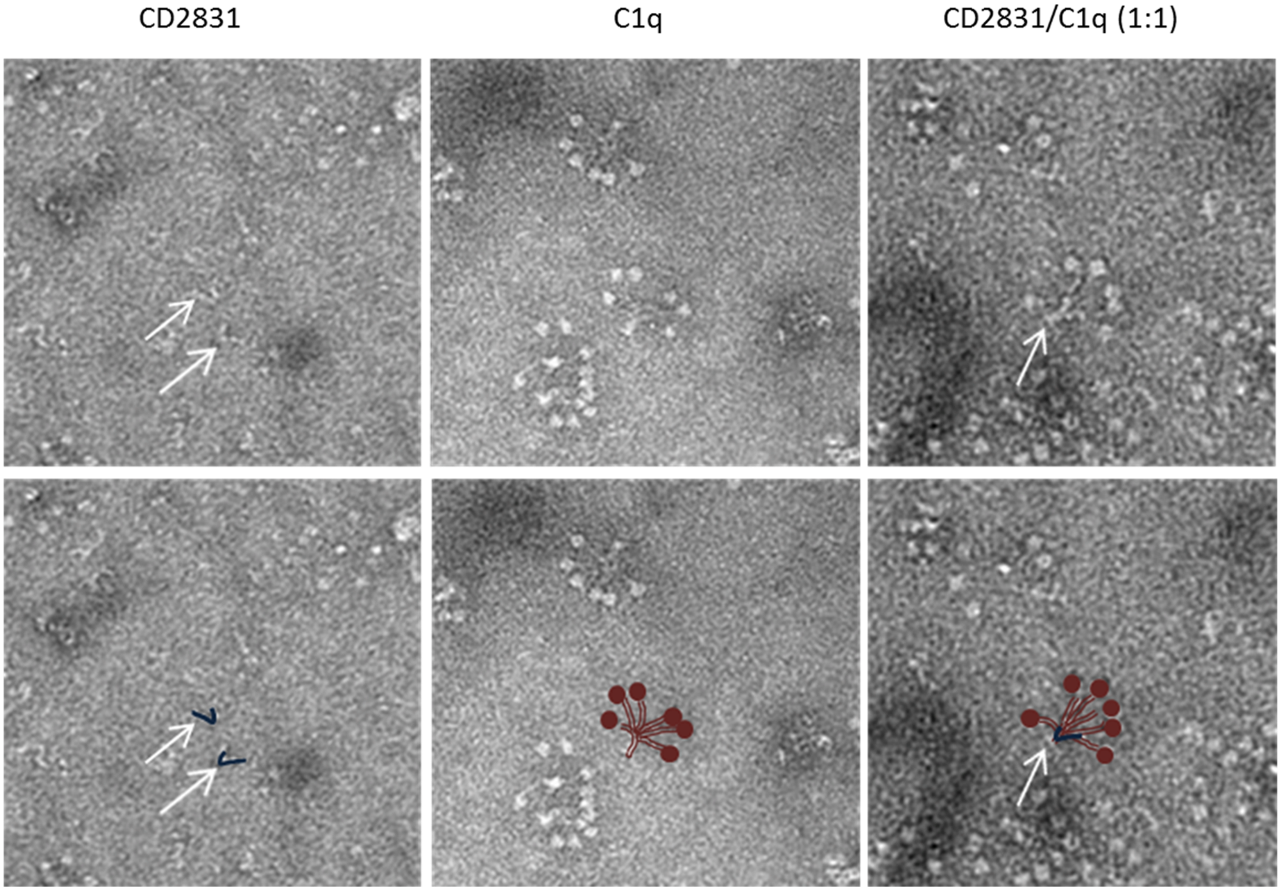

Fig. S3 CD2831 associates to C1q stalk

Transmission Electron Microscopy images showing CD2831 (white arrows), human C1q and CD2831/C1q in 1:1 molar ratio. The lower panels show drawings of CD2831 (blue) interacting with the stalk region of C1q (red). Purified CD2831 and/or C1q were spotted on a 300-mesh copper TEM grid (Agar Scientific, Stansted, UK). After 1 minute of adsorption the excess was blotted with Whatman filter paper and the samples were counterstained with Nano-W (Nanoprobes, NY, USA) for 30 seconds. The images were acquired at 120000x magnification using a TEM FEI Tecnai G2 spirit microscope.

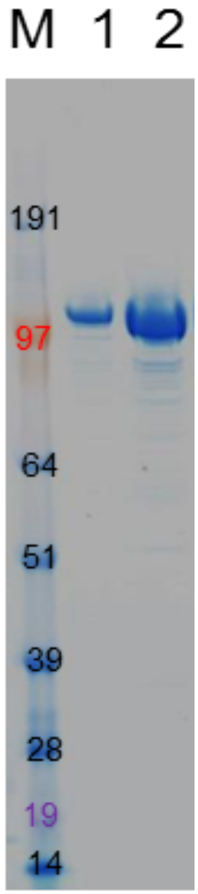

Fig. S4   SDS-PAGE gel of recombinant CD2831 full-length

SDS-Page and Coomassie blue staining of 50 ng (lane 1) and 100 ng (lane 2) of the purified full-length recombinant CD2831 protein (108 kDa).
